# Supplementary figures and images for: Gene Expression Divergence in Eugenia uniflora Highlights Adaptation across Contrasting Atlantic Forest Ecosystems
Source: Plants (Basel). 2024 Sep 28;13(19):2719. doi: 10.3390/plants13192719 (PMC11478965; doi:10.3390/plants13192719)

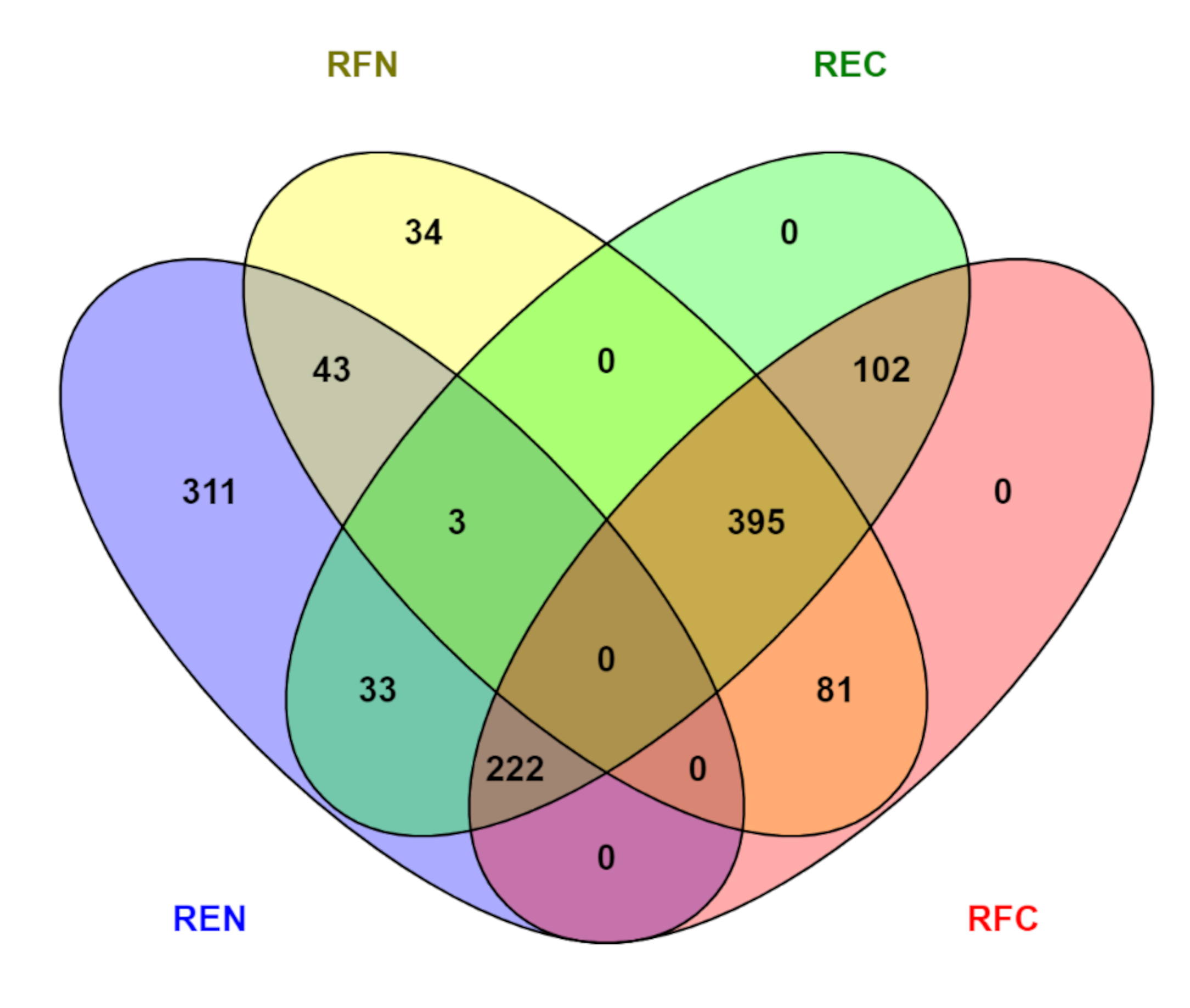

Supplement: Supplementary file 1 [file plants-13-02719-s001.zip › Figure S1.tiff]
